# Zinner's syndrome: A rare diagnosis of congenital seminal vesicle cyst and renal agenesis on basis of radiological imaging and its management

**DOI:** 10.1016/j.ijscr.2022.107434

**Published:** 2022-07-21

**Authors:** Aavishkar Raj Regmi, Sarmendra Mishra, Adarsh Gurung, Archana Acharya, Laxman Dutta Paneru, Ajnish Ghimire

**Affiliations:** a Department of Surgery, KIST Medical College, Imadol, Lalitpur, Nepal; b Nepal Health Research Council, Ram Shah Path, Kathmandu, Nepal

**Keywords:** Case report, Zinner's syndrome, Seminal vesicle cyst, Renal agenesis, Urology

## Abstract

**Introduction and importance:**

Seminal vesical cyst (SVC) together with ipsilateral renal agenesis known as Zinner's syndrome is a rare congenital malformation mostly asymptomatic and is detected in second to fourth decade of life presenting with symptoms of bowel and bladder neck irritation/obstruction. Diagnosis is based mainly on various imaging techniques with MRI being the confirmatory. Recent management includes laparoscopic excision of the SVC but conventional aspiration of the cystic fluid together with explorative open excision of the cyst is still regarded useful.

**Case presentation:**

A 32 years old male presented with urgency and frequency of micturition, constipation, post ejaculatory pain and USG, CT, MRI findings suggestive of right renal agenesis and bilateral seminal vesicle cyst.

**Clinical discussion:**

As other studies show, our patient is a sexually active male with characteristic symptoms of bladder and bowel obstruction. USG, CT, MRI were used for diagnosis co-relating other studies. Intervention was done by aspiration of the cystic fluid and open surgical excision of the cyst.

**Conclusion:**

Zinner's Syndrome is uncommon cause of symptoms of bowel and bladder obstruction in young men; whose diagnosis is mostly based on imaging techniques.

## Highlights

• Seminal vesical cyst (SVC) together with ipsilateral renal agenesis known as Zinner’s syndrome is rare.
• It is detected in second to fourth decade of life presenting with symptoms of bowel and bladder neck irritation/obstruction.
• Diagnosis is based mainly on various imaging techniques with MRI being the confirmatory.
• Conventional aspiration of the cystic fluid together with explorative excision of the cyst is still regarded useful.

## 1 Background

Seminal vesicle cyst (SVC) is one of the extremely rare entity, which occurs in 0.005 % of the population [1]. Zinner's syndrome (ZS) is one of the rare congenital anomaly of the seminal vesicle and ipsilateral upper urinary tract which is characterized by the simultaneous presence of unilateral renal agenesis, ipsilateral seminal vesicle cyst, and ipsilateral ejaculatory duct obstruction (EDO) [2], [3], [4], [5]. EDO causes seminal fluid accumulation and then to enlarged seminal vesicles [6]. The first case of SVC along with ipsilateral renal agenesis was reported by Zinner in 1914, and anomalies associated have been mentioned in the literature [7].

Patients may remain asymptomatic and are diagnosed coincidentally [8]. If symptomatic, they may present with common symptoms that include perineal, micturition, and ejaculatory pain, pollakisuria, dysuria, constipation, increased urinary frequency, hematuria, hematospermia, urinary tract infection, symptoms of epididymitis and prostitis including infertility and sometimes anuresis [7], [9], [10], [11], [12], [13], [14].

## 2 Case presentation

We report the case of a 32 years old male, father of a 4 years old son, working as a carpenter, who presented to the surgery outpatient department of a tertiary level hospital in Nepal, with a 2 year history of urgency, increased frequency of micturition, constipation. However, the symptoms increased over time and there was perianal pain for 2 months, pain after ejaculation for 1 month. The International Prostatic Symptom Score was 14. The patient's history was negative for fever, hematuria, hematospermia, per rectal bleeding. There were no any comorbidities and significant family history. The patient was not under any medications.

Findings on his arrival were as follows: body height, 172 cm; body weight, 66 kg - BMI was 22.3 (normal weight).; blood pressure, 120/70 mmHg; pulse, 80 beats/min; body temperature, 37.3 °C; no anemia and icterus in the palpebral conjunctiva; no edema in the legs, no cyanosis; no any palpable lymph nodes breathing sounds were clear/no secondary noises; no heart murmurs. No any other significant examination findings were present per abdomen. Per rectal examination revealed an anal fissure measuring 0.5 cm × 0.2 cm at 5 o'clock position.

Hematological tests were sent and urinalysis was performed, the report of which were within normal limits. Urine culture also didn't show growth of any organism.

**Fig. 1:**
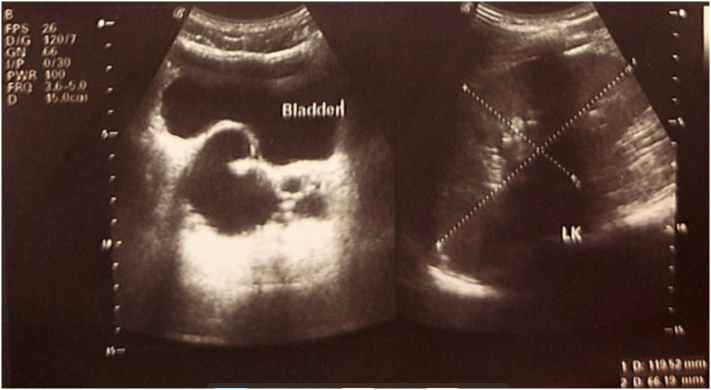
USG abdomen and pelvis: retrovesicular cystic lesion.

**Fig. 2:**
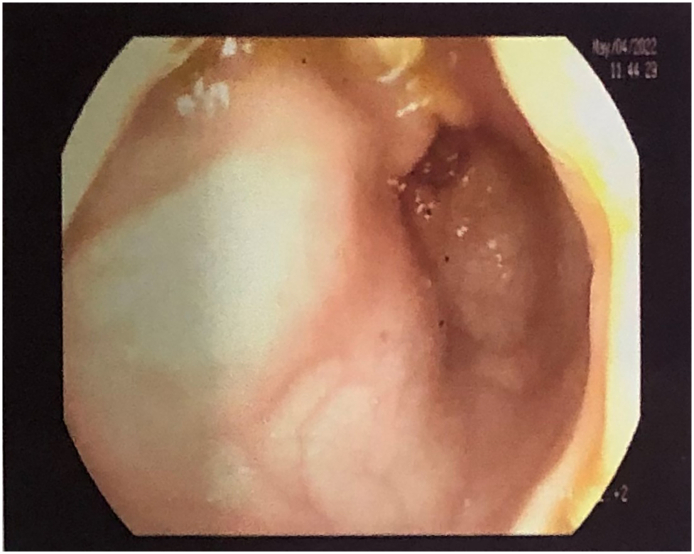
Sigmoidoscopy: globular submucosal lesion in the rectum.

Ultrasonography (USG) of Abdomen and Pelvis (Fig. 1) couldn't find the right kidney and revealed few cystic lesion in the right pelvis close to prostate suggestive of dilated seminal vesicle, diffuse echogenic debris in urinary bladder suggestive of cystitis with normal sized prostate weighing approximately 11 g with normal outline and echo pattern. The patient was then managed conservatively with oral antibiotics, disodium hydrogen citrate, aceclofenac and selective antagonist of post-synaptic alpha-adrenoreceptors and with ointment nitroglycerine and isphagulla. The patient's symptoms were still not relieved for which a sigmoidoscopy (Fig. 2) was done as further diagnostic procedure which showed a globular submucosal lesion in the rectum. However, biopsy was not taken.

**Fig. 3:**
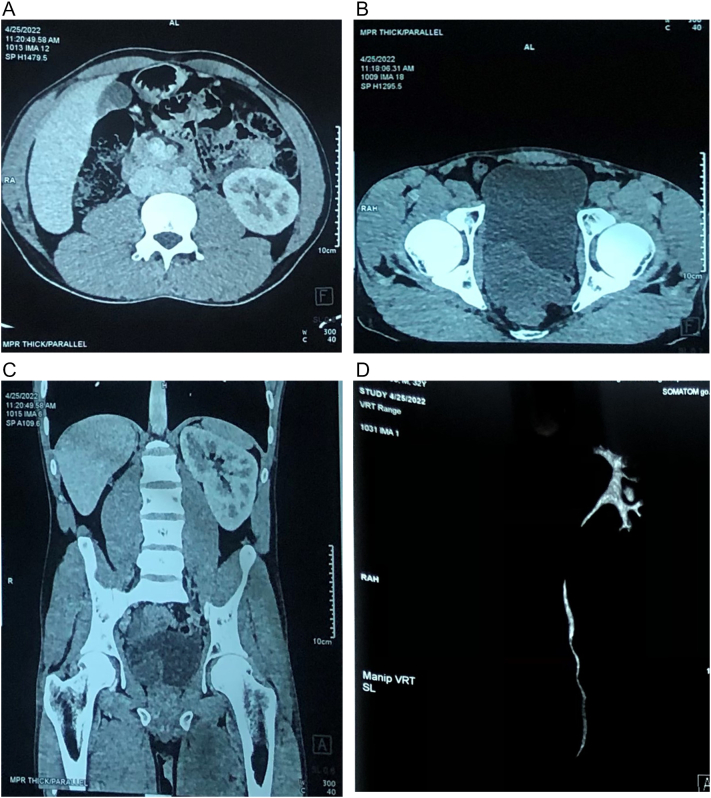
A: CT abdomen and pelvis: no evidence of the right renal unit. B: CT abdomen and pelvis: presence of a non-enhancing cystic lesion. C: CT abdomen and pelvis no evidence of the right renal unit with the presence of a non-enhancing cystic lesion. D: CT urogram: absent right kidney and right ureter.

Computed Tomography (CT) of Abdomen and Pelvis (Fig. 3A-D) were done which revealed solitary, left, functioning kidney, with no evidence of the right renal unit with the presence of a non-enhancing cystic lesion measuring 69 × 71 mm in recto sigmoid region more on the right side of the pelvis posterior to the urinary bladder. The seminal vesicles bilaterally were found to be enlarged. Post contrast study showed no enhancement, no enhancing septa with the lesion showing narrow tubular structure with blind area in proximal part. Post CT USG showed bean shaped cystic lesion in the posterior part of urinary bladder with significant post void urine (93 ml).

**Fig. 4:**
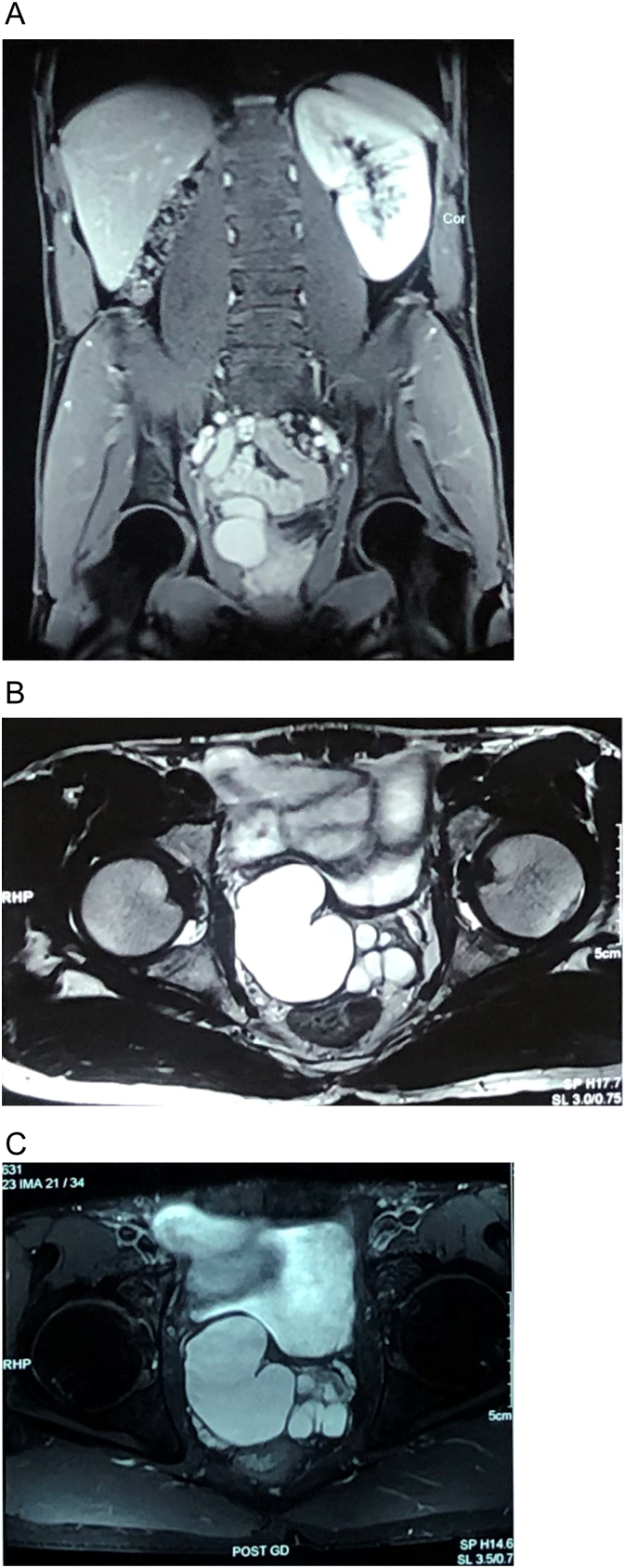
A: MRI abdomen and pelvis, T1, coronal. B: MRI abdomen and pelvis, T1, transverse. C: MRI abdomen and pelvis, oblique.

An MRI abdomen and pelvis (Fig. 4A-C) was also done which showed:

i. Dilated lobular and tortuous cystic lesion in the Right side of pelvic cavity, the maximum diameter of which was 4 cm, extending along the right side pelvic wall up to the level of aortic bifurcation and opening into the prostatic urethra and showing wall enhancement in post contrast images. The lesion was seen to be abutting prostate inferiorly, urinary bladder and small bowel loops anteriorly
ii. Another 4.6 × 3.1 × 2.5 cm sized similar dilated, tubular and tortuous cystic lesion in the left side of pelvic cavity, with diameter of 1.4 cm, opening in prostatic urethra. Prostate normal in size, outline and parenchymal signal intensity.
iii. Non-visualization of Right kidney. Left kidney measuring 12.5 × 6.7 cm in size However, MRI did not reveal any rectal mass or wall thickening of the rectum.

Urethrocystoscopy was done which showed no any lesions in urethra or bladder with a protruding mass at right lateral trigonal region which was partially obstructing the bladder neck. Imaging findings along with cystoscopic examination lead to the diagnosis of Zinner's Syndrome. Pelvic exploration with aspiration of cystic fluid accompanied by open surgical excision of cystic structure was then done by the consultant urologist. Operative finding include enlarged bilateral seminal vesicles, right sided about 5 cm in diameter left sided around 2 m in diameter; with 70 ml of cystic fluid in the right seminal vesicle. The aspirated cystic fluid was sent for microbiological examination which eventually came negative while the excised tissue was sent for histopathological assessment which revealed cyst wall lined by pseudostratified columnar epithelium. Few of the epithelial cells show reddish brown lipofuscin granules in the cytoplasm. Wall of the cyst comprises of fibromuscular tissue with chronic inflammatory cell infiltrates. These findings are consistent with Seminal Vesicle Cyst.

The post-operative period was uneventful and the patient did not experience any further genitourinary discomfort. The patient was then discharged on 3rd post-operative day and was symptomatically better and hemodynamically stable at time of discharge.

## 3 Discussion

Unilateral renal agenesis is found to be present in 0.1 % of newly born babies [15]. 12 % of men having unilateral renal agenesis are also associated with genitourinary anomalies [10]. In 68 %, SVC is linked with ipsilateral renal agenesis [10]. As both seminal vesicle and kidney arise from the mesonephric (Wolffian) duct at the time of embryogenesis, the association of congenital SVC and renal agenesis of same side together is common [11]. In our case, bilateral SVC is present which is associated with right sided renal agenesis. ZS is diagnosed in the 2nd to 4th decades of patient's life [7], as in our case.

SVC with size smaller than 5 cm in diameter remains asymptomatic and usually are discovered incidentally [13], [16]. Symptoms are usually seen during the period of high sexual and reproductive activity [17]. This relates to our case, as our case had both right and left sided cystic lesion which were 4.0 cm and 1.4 cm in diameter respectively and was asymptomatic for first 30 years of his life. Regarding the presentation of the patient in our case, he had symptoms characterizing bladder outlet and bowel obstruction without features of genitourinary infection. Fertility was preserved in our patient.

Imaging is key to diagnosis. Transabdominal Sonography [18], Computed Tomography (CT) [2] and MRI [5] are considered important in the radiological diagnosis and surgical planning, as in our case.

Widely accepted treatment methods include open exploration with vesiculectomy, transperitoneal or transrectal aspiration of the cyst, or transurethral deroofing of the cyst [7], [11], [19]. Pelvic exploration, aspiration of the cystic fluid and open surgical excision of the SVC together, as done in our case, can be considered the definitive management, as simple aspiration has been seen to be complicated by recurrence of cyst and infection [20].

Recent techniques include laparoscopy (transperitoneal approach), as reported by Seo et al. which is minimally invasive as compared to the conventional open surgery which in very invasive due to deep location of seminal vesicles in retrovesical area [12].

USG can be the imaging of choice for follow up in patients with asymptomatic SVC as it is cheap, available, noninvasive and can be easily done by the urologist alone [7].

## 4 Conclusion

Zinner's Syndrome is uncommon cause of symptoms of bowel and bladder obstruction in young men; whose diagnosis is mostly based on imaging techniques. USG is valuable in initial steps as well as follow up, CT scan defines the origin of cyst and MRI is important for diagnosis and surgical planning. Aspiration alone is not the treatment of choice, but being added with open surgical excision can be proven reliable. However, newer laparoscopies techniques can be used.

## Patient perspective

The patient was not aware of his condition before he visited our Centre. The patient was glad to be diagnosed and was satisfied by the treatment.

## Ethical approval

Ethical approval was not needed for this study according to local/national guidelines.

## Consent

Written informed consent was obtained from the patient for publication of this case report and accompanying images. A copy of the written consent is available for review by the Editor-in-Chief of this journal on request.

## Guidelines

This work has been completed in line with the SCARE criteria [21].

## Funding sources

No any grant was received for the preparation and publication of this manuscript from funding agencies in the public, commercial, or not-for-profit sectors.

## Author contributions

Aavishkar Raj Regmi, Sarmendra Mishra conceptualized the study and reviewed the topic. Aavishkar Raj Regmi, Adarsh Gurung and Archana Acharya wrote the original draft. Aavishkar Raj Regmi and Adarsh Gurung edited the manuscript. Sarmendra Mishra and Ajnish Ghimire reviewed the manuscript. Aavishkar Raj Regmi and Laxman Dutta Paneru worked up with publication.

## Registration of research studies

Not applicable.

## Guarantor

Aavishkar Raj Regmi accepts full responsibility for the work and/or the conduct of the study, had access to the data, and controlled the decision to publish.

## Provenance and peer review

Not commissioned, externally peer-reviewed.

## Declaration of competing interest

The authors have no any conflicts of interest to declare.
